# Morphology, bioacoustics, and ecology of *Tibicen neomexicensis* sp. n., a new species of cicada from the Sacramento Mountains in New Mexico, U.S.A. (Hemiptera, Cicadidae, *Tibicen*)

**DOI:** 10.3897/zookeys.337.5950

**Published:** 2013-10-01

**Authors:** Brian J. Stucky

**Affiliations:** 1 Department of Ecology and Evolutionary Biology, University of Colorado, Boulder, Colorado, USA

**Keywords:** Cicadidae, Tibicen, bioacoustics, cicada, cryptic species

## Abstract

*Tibicen neomexicensis* **sp. n.**, a new species of cicada found in the Sacramento Mountains of southcentral New Mexico, is described. *Tibicen neomexicensis* closely resembles *Tibicen chiricahua* Davis morphologically, but males of the two species have highly distinct calling songs that differ in phrasal structure, amplitude burst rates, and pulse structure. Unlike *Tibicen chiricahua*, male *Tibicen neomexicensis* use conspicuous dorso-ventral abdominal movements to modulate the amplitude and frequency of their calls. *Tibicen neomexicensis* is also smaller on average than *Tibicen chiricahua*, and differences in the color patterns of the wing venation identify these two species morphologically. Both species are dependent on pinyon-juniper woodlands and have similar emergence phenologies. These species appear to be allopatric, with *Tibicen chiricahua* found west of the Rio Grande in New Mexico, Arizona, and Mexico, and *Tibicen neomexicensis* so far known only from New Mexico, east of the Rio Grande. *Tibicen chiricahua* and *Tibicen neomexicensis* males share a common genitalic structure that separates them from all other species of *Tibicen*, and the possible evolutionary and biogeographic history of these likely sister species is also discussed.

## Introduction

Cicadas, crickets, katydids, and many other insects produce airborne acoustic signals that play an essential role in reproduction (Alexander 1960, 1967, Capinera et al. 2004). For cicadas, acoustic communication is the single most important factor in mate recognition, pair formation, and premating reproductive isolation (Alexander and Moore 1958, Boulard 2006). As such, the calling songs of male cicadas have become an essential part of cicada taxonomy. Acoustic studies have led to the discovery of numerous “cryptic” cicada species that are morphologically nearly identical to other species but can be readily identified by their unique mating calls (e.g., Davis 1922, Alexander and Moore 1962, Popov 1989, Marshall and Cooley 2000, Quartau and Simoes 2005, Sueur and Puissant 2007, Cole 2008, Gogala et al. 2008). In some cases, acoustic analyses provide the only means to identify a species with certainty (e.g., Sueur et al. 2007, Gogala et al. 2008).

During fieldwork in New Mexico in 2012, I observed cicadas that fit the morphological description of *Tibicen chiricahua* Davis (Davis 1923) in both the Magdalena Mountains of west-central New Mexico and the Sacramento Mountains of southcentral New Mexico. However, the populations from these two mountain ranges had completely different calling songs, suggesting the presence of two species and rendering the taxonomic identities of both populations uncertain. To help resolve this problem, I traveled to the type locality of *Tibicen chiricahua*, Pinery Canyon in the Chiricahua Mountains of southeastern Arizona (Davis 1923), to record the calls of true *Tibicen chiricahua*. The calling songs recorded in the Chiricahua Mountains were the same as those recorded in the Magdalena Mountains in New Mexico, revealing that the cicadas in the Sacramento Mountains were a previously unrecognized species, described here as *Tibicen neomexicensis*. Upon closer inspection, it became clear that these two species exhibited subtle morphological differences, as well.

In this paper, I describe *Tibicen neomexicensis* and compare its morphology to *Tibicen chiricahua*, describe and compare the calling songs and calling behaviors of *Tibicen neomexicensis* and *Tibicen chiricahua*, and compare the geographic distributions of the two species. Finally, I discuss the general ecology, phenology, and daily activity patterns of *Tibicen neomexicensis* and consider its possible evolutionary relationship with *Tibicen chiricahua*.

## Methods

### Field sites and specimens examined

All field work was conducted during May and June of 2012. Cicadas identified as *Tibicen chiricahua* were observed and audio recorded in the Magdalena Mountains west of Socorro, New Mexico, and at the type locality for *Tibicen chiricahua*, Pinery Canyon in the Chiricahua Mountains of southeastern Arizona (Davis 1923). Specimens of the new species were observed and recorded at its type locality.

To estimate the geographic ranges of the two species and better understand morphological variation across these ranges, I examined a total of 202 specimens previously identified as *Tibicen chiricahua* from the collections of the Arthropod Museum at New Mexico State University (NMSU), the C. P. Gillette Museum of Arthropod Diversity at Colorado State University (CSUC), the Frank M. Hasbrouck Insect Collection at Arizona State University (ASUT), the Snow Entomological Museum at the University of Kansas (SEMC), the Texas A&M University Insect Collection (TAMU), the University of Arizona Insect Collection (UAIC), and the University of Colorado Museum of Natural History (UCMC). The SEMC specimens included a male paratype of *Tibicen chiricahua* from Davis’s original type series. I also examined high-resolution digital photographs of the holotype male and allotype female of *Tibicen chiricahua*, which are currently housed in the collection of the Academy of Natural Sciences of Drexel University (ANSP).

### Morphology

Morphological terminology follows Moulds (2005, 2012). Morphometric measurements were made with a digital caliper. Fore wing width was measured from the node to the posterior edge, head width was measured between the eyes, and pronotum width was taken at the widest point between the lateral angles.

### Audio recordings and analysis

Cicada calling songs were recorded in the field using a Sennheiser ME 66 shotgun microphone with an MZW 66 PRO windscreen connected to a Sony PCMM10 digital audio recorder. All recordings were made as uncompressed, 16-bit PCM audio at a sampling rate of 44.1 kHz. For each recording, the microphone was held between 0.5 and 2 meters away from the calling cicada. This was close enough to minimize background noise, but far enough away to avoid any near-field acoustic effects in the frequency range of the calling songs (Michelsen and Nocke 1974, Peterson 1980).

Cicada calls were analyzed to determine peak frequencies, amplitude burst rates, and the number of sound pulses per amplitude burst. In this paper, I use the term “pulse” in the sense of Broughton (1963) and “amplitude burst” to mean a single group of high-amplitude pulses in an amplitude-modulated pulse train (see Figures 4 and 5). I elected to use “amplitude burst” rather than “syllable,” which has been used inconsistently in cicada bioacoustics and usually with disregard to the precise definitions of Broughton (1963) and Ragge and Reynolds (1998).

Analyses were conducted using Audacity® (Audacity Team 2012) and custom-written software. Peak frequency was estimated by identifying the highest peak in a power spectral density plot generated by a 512-sample Fast Fourier Transform with the Hamming window function. If there were two or more peak frequencies that differed by less than 0.5 dB, their average was taken as the overall peak frequency. The amplitude burst rate (i.e., pulse amplitude modulation rate) was calculated by first estimating the call’s amplitude envelope, then using a gate function to identify the amplitude peaks in the signal (Beeman 1998). To estimate the number of sound pulses per amplitude burst, a sequence of 12 bursts was selected from the middle of each call, the audio data were normalized so that the maximum signal amplitude was at 0 dBFS, and the beginning and ending pulses of each amplitude burst were determined by identifying the first and last pulses with absolute sample values that exceeded 50% of the maximum sample value (that is, -6.02 dBFS).

The calls of both *Tibicen chiricahua* and *Tibicen neomexicensis* can be divided into three phrases (see results below), but the boundaries between phrases are often indistinct. To avoid the non-repeatability and potential bias of estimating the phrase durations by simple visual or aural inspection of the call oscillograms, I used objective criteria based directly on the audio data. All audio data were first normalized so that the peak amplitude was at 0 dBFS. For both *Tibicen chiricahua* and *Tibicen neomexicensis*, the first phrase began at the start of the call, and the end of the first phrase and beginning of the second phrase was defined by the first amplitude burst that reached -3 dBFS. For *Tibicen chiricahua*, the end of the second phrase was defined by the last amplitude burst to reach -3 dBFS, while for *Tibicen neomexicensis*, the end of the second phrase was defined as the end of the modulated portion of the call. For both species, the third phrase consisted of all audio from the end of the second phrase to call termination.

I did not include ambient air temperatures in the acoustic analyses. North American cicadas utilize a variety of behavioral and physiological thermoregulation tactics, so ambient temperature is often a poor indicator of a calling cicada’s body temperature (Toolson 1987, Hastings 1989, Sanborn et al. 1992, Sanborn 2000, 2004).

### Biogeography

The locations of field sites that I personally visited were determined using a Garmin nüvi 260 GPS receiver. Specimen label data lacking latitude and longitude information were georeferenced primarily using data from the Geographic Names Information System of the United States Geological Survey (http://geonames.usgs.gov/), and in some cases using Google Earth (http://earth.google.com/). Landcover data were from the Southwest Regional Gap Analysis Project (USGS National Gap Analysis Program 2004). The distribution of pinyon-juniper woodlands was estimated by mapping all land cover types that included both pinyon pines (*Pinus edulis*, *Pinus monophylla*) and junipers (*Juniperus* spp.) as dominant tree or shrub species (codes S038, S039, S040, S052, and S112). Landcover types with junipers but not pinyon pines and sparsely vegetated types (< 10% plant cover) were excluded. QuantumGIS (Quantum GIS Development Team 2012) was used to produce the distribution map.

### Statistical analysis

Acoustic and morphometric data were analyzed in R (R Core Team 2013) using univariate multiple linear regression with categorical predictor variables (i.e., ANOVA models). Morphometric data were modeled with species and sex as predictors, while acoustic data were modeled with species as the sole predictor. For all analyses, preliminary *F*-tests were used to compare models with locality as a predictor (two localities for *Tibicen chiricahua* and the type locality for *Tibicen neomexicensis*) to models that grouped all *Tibicen chiricahua* data together (i.e., used species as a predictor). In all cases, there was not a significant difference between the models (all *p*-values > 0.0788), so the data from the two locations for *Tibicen chiricahua* were grouped together for both the morphometric and acoustic analyses. Plots of the standardized residuals were examined to verify that the data met the model assumptions. Because *Tibicen neomexicensis* is most easily separated from *Tibicen chiricahua* by its distinctive calling song, the statistical analyses only included specimens from localities that had been acoustically surveyed.

## Results

### Tibicen neomexicensis sp. n.

http://zoobank.org/4847B3E5-22BF-4262-868B-FA06341DB9B4

http://species-id.net/wiki/Tibicen_neomexicensis

#### Type locality.

USA, New Mexico, Lincoln County, Lincoln National Forest, near the junction of Forest Road 105 and State Highway 37, 33.5287°N, 105.6939°W (datum: WGS84), elevation 2188 m, pinyon-juniper forest.

**Figure 1.**
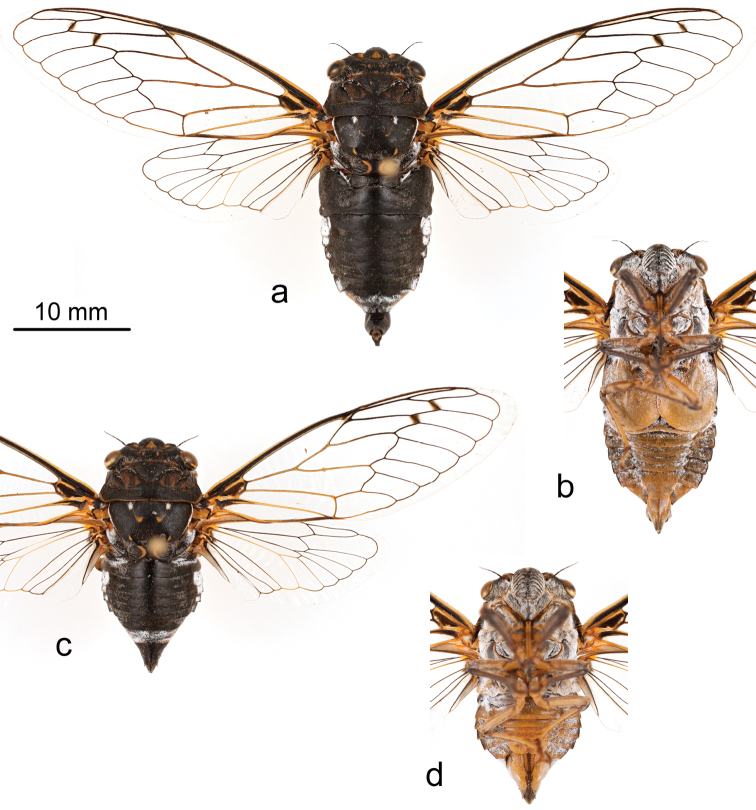
Holotype male of *Tibicen neomexicensis* sp. n.: **a** dorsal view **b** ventral view; and paratype female: **c** dorsal view **d** ventral view.

**Figure 2.**
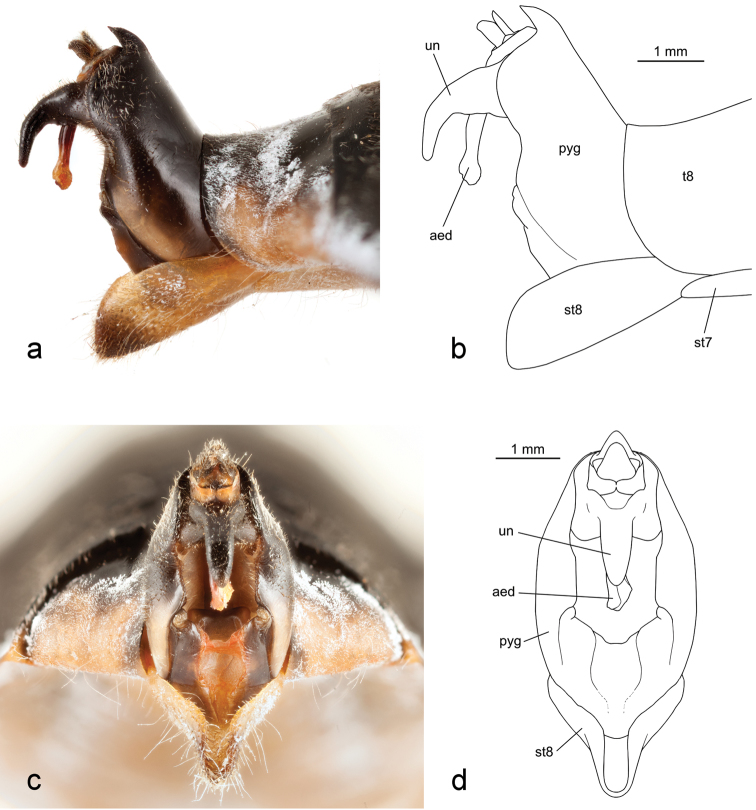
Terminalia of holotype male *Tibicen neomexicensis* sp. n.: **a** and **b** lateral view; and **c** and **d** posterior view. Abbreviations: **aed**–aedeagus, **pyg**–pygofer, **st**–sternite, **t**–tergite, **un**–uncus.

**Figure 3.**
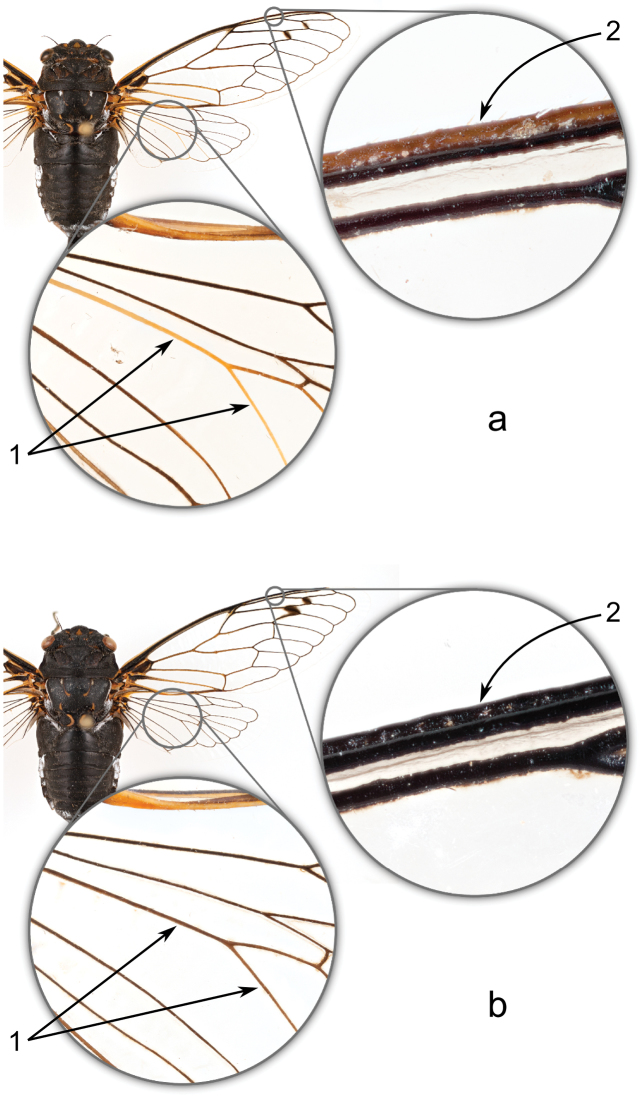
Key morphological features separating *Tibicen neomexicensis* sp. n. (**a**) and *Tibicen chiricahua* (**b**): **1**) the color of the cubitus anterior vein (CuA) and its second branch (CuA_2_) in the hind wing, and **2**) the color of the anterior margin of the subcostal vein (Sc) of the fore wing.

**Holotype male.** Pinned specimen (Figures 1–3). Original label: “NM: Lincoln Co. | Lincoln NF, FR 105 | 33.5287°N, 105.6939°W | May 31, 2012 7178 feet | Brian and Erin Stucky”. UCMC, specimen identifier UCMC 0046172.

**Paratypes.** 8 males and 3 females, same label data as holotype; 2 males and 2 females, same label data as holotype except collected on May 30, 2012. The paratypes are currently housed in the UCMC and the author’s collection. Upon publication, paratypes will also be transferred to the ANSP, the Smithsonian National Museum of Natural History (NMNH), NMSU, and the SEMC.

#### Description.

*Head*. Slightly wider than anterior margin of pronotum. Vertex and frons black, marked with orange-brown on the posterior margin near the eyes and immediately lateral of the lateral ocelli. Supra-antennal plates black dorsally with an orange-brown mark adjacent to the postclypeus, orange-brown ventrally marked with black immediately above the antennae, and orange-brown along the anterior margin except for immediately adjacent to the postclypeus. Antennae mostly black with distal margin of scape yellowish, proximal half of pedicel dark brown in some specimens. Dorsal surface of head sparsely covered with short golden hairs and with longer, silvery-white hairs behind the eyes. Ventral surface mostly covered with dense, silvery-white hairs. Postclypeus black, marked with orange-brown on the anterior-medial margin and with a triangular orange-brown mark adjacent to the frontoclypeal suture. Transverse grooves of postclypeus lined with pruinosity and silvery-white hairs. Anteclypeus black, yellowish posterolaterally, with a medial brown spot at the junction with the postclypeus. Lora mostly black, marked with yellow along the lateral margins. Genae black anteriorly, yellowish posteriorly where they border the lora. Proximal two thirds of rostrum yellowish, labrum and distal one third of rostrum black, with the apex extending posteriorly to the hind coxae.

*Thorax*. Pronotum black, marked faintly with dark brown between the paramedian and lateral fissures and between the lateral fissures and pronotal collar, brown markings often more extensive in females. Pronotal collar black, lined with orange along the anterior margin between the eyes and along the lateral margins, extending to the posterior margin and fading to black medially. Some specimens have the entire posterior margin lined with orange. Pronotum sparsely covered with fine golden hairs. Mesonotum black marked with orange as follows: two J-shaped lines following the parapsidal suture, a small spot at the terminal end of each anterior arm of the cruciform elevation, two C-shaped marks starting at the origin of the anterior arms of the cruciform elevation and curving medially then laterally towards the posterior arms, and a large mark near the base of each fore wing. Mesonotum with two small pruinose spots on the anterior margin just lateral of the parapsidal sutures, lateral margin also pruinose. Mesonotum sparsely covered with fine golden hairs, with longer silvery-white hairs in the depressions of the cruciform elevation and along the posterolateral margins. Visible portion of metanotum black, covered with silvery-white hairs laterally. Ventral surface of thorax often heavily pruinose and covered with silvery-white hairs, yellowish except for katepisternum 2, anterior portion of basisternum 2, anepimeron 2, central part of katepimeron 2, meron 2, anterior portions of trochantins 2 and 3, episternum 3, and basisternum 3, all of which are black.

*Legs*. Fore coxae orange marked with brown apically and with the anterolateral surface dark brown except along the margins. Middle and hind coxae orange marked with dark brown laterally. Coxae covered with silvery-white hairs and often pruinose. Trochanters orange, variably marked with brown. Femora orange, apex mostly yellow, brown ventrally, with longitudinal brown stripes that often merge apically and basally. Silvery-white hairs on femora mostly confined to brown markings. Femoral spines brown basally with dark brown apices. Tibiae orange ventrally, brown dorsally with brown markings expanded at the base, covered with silvery-white hairs. Tibial spurs and comb dark brown. Tarsi variable in color but usually dark brown dorsally and light brown to orange ventrally. Claws brown basally with dark brown apices.

*Wings*. Fore wings hyaline with 8 apical cells, crossveins r and r-m usually strongly infuscated. Costal margin yellow, C vein black, R+Sc vein black with posterior margin pale along the radial cell. Sc vein black beyond the node, subcostal margin brown to dark yellow. Basal cell mostly black, anterior and posterior borders yellow. M vein yellowish-black from its base to the junction with M_1+2_, black beyond. M_3+4_ yellowish-black. M_1+2_ yellowish-black becoming black apically. CuA vein yellow from its base to the junction with CuA_2_, yellowish-black beyond. CuA_2_ yellowish-black. CuP+1A and 2A+3A veins mostly yellow, ambient vein dark yellowish-black, remaining venation black. Hind wings hyaline with 6 apical cells. Sc+RA, RA, CuA between base and CuA_2_, and CuA_2_ veins mostly yellow to yellowish-orange. CuA between CuA_2_ and m-cu, and CuA_1_ veins yellow to yellowish-black. Ambient vein black marked with yellow along 1st cubital cell and 6th apical cell. Remaining venation mostly black or dark brown. 3rd anal cell gray marked with reddish-orange basally.

*Opercula*. Male opercula yellowish marked with black on the anterolateral and anteromedial margins, overlapping medially. Posterior margins smoothly rounded, not quite reaching the posterior margin of sternite II. Female opercula yellowish, becoming black anterolaterally. Posterior margin sinuate, reaching the anterior margin of sternite II. Meracanthus black basally with a yellow apex.

*Abdomen*. Dorsal surface of abdomen almost entirely black, sparsely covered with short golden and silvery hairs. Tergite 8 orange-brown laterally. Tergites 3-7 often marked with orange-brown laterally, markings usually strongest on tergite 3. Timbal covers black, sometimes dark brown centrally, completely concealing timbal. Timbal with 3 long ribs, 4 intercalary ribs, and an incomplete 4th long rib. Dorsal abdomen pruinose at the following locations: along the anteromedial margins of the timbal covers in males; along the anterolateral margins of tergite 2 in females; the lateral margins of tergites 3-7, most prominently on tergite 3; the lateral margins of tergite 8, often extending medially to cover most of the tergite. Sternites orange to yellowish, usually dark brown laterally and anterolaterally. Epipleurites orange to yellowish, indistinctly marked with dark brown or black. Ventroposterior portion of male sternite VIII dark brown.

*Male terminalia*. Pygofer black, becoming brown or yellowish laterally along the lobes, and with a small brown spot dorsally at the base of the dorsal beak. Dorsal beak not quite as long as anal styles. Anal styles black. Median lobe of uncus slender, black, strongly bent ventrally and terminating in a rounded point. Aedeagus reddish-brown.

*Female terminalia*. Abdominal segment 9 yellowish-orange ventrally, black dorsally starting at about the lateral mid-line. Dorsal beak about as long as anal styles. Sternite VII yellowish-orange, usually brown laterally, deeply notched at the middle of the posterior margin. Visible portion of gonocoxite IX yellowish-orange, indistinctly marked with brown near the posterior end. Ovipositor sheath black, ventromedial margins partially lined with orange. Ovipositor sheath extends posteriorly about as far as anal styles.

*Measurements*. All measurements are reported in mm as mean (range, standard deviation). Males (*n* = 11): head width: 8.3 (8.1–8.5, 0.13); pronotum width: 8.9 (8.5–9.3, 0.25); fore wing length: 28.1 (26.8–29.6, 0.76); fore wing width: 9.8 (9.3–10.5, 0.41); body length: 24.9 (23.1–26.3, 1.05). Females (*n* = 5): head width: 7.8 (7.7–7.9, 0.07); pronotum width: 8.4 (8.2–8.5, 0.14); fore wing length: 26.7 (26.2–27.4, 0.48); fore wing width: 9.1 (8.9–9.3, 0.20); body length: 20.0 (19.3–20.9, 0.64).

#### Etymology.

The specific epithet refers to the U.S. state of New Mexico. As far as is currently known, *Tibicen neomexicensis* is endemic to this state.

##### Morphometric comparison of *Tibicen neomexicensis* and *Tibicen chiricahua*

Five morphometric measurements were taken for both *Tibicen chiricahua* and *Tibicen neomexicensis*: fore wing length, fore wing width, head width, pronotum width, and total body length. The correlation coefficient matrix for these five variables revealed that all five measurements were very strongly correlated with one another. All pairwise correlation coefficients excluding body length were > 0.91, and all pairwise correlation coefficients including body length were > 0.80. Body length in adult cicadas is not constant and instead varies according to a cicada’s abdominal posture, so the lower correlation coefficients for body length were not surprising. Given the high correlation among the five variables, analyzing each separately would have been largely redundant, so comparative analysis focused on fore wing length (Table 1). Fore wing length is invariant in adult cicadas and easily measured for either live or preserved specimens.

**Table 1.** Summary statistics for fore wing length and six acoustic variables for *Tibicen neomexicensis* sp. n. and *Tibicen chiricahua* (M = male, F = female).<br/>

**variable**	**species**	**mean**	**95% CI**	**range**	**std. dev.**	***n***
fore wing length (mm)	*Tibicen chiricahua*, M	31.3	30.6–32.0	29.4–33.1	1.06	11
	*Tibicen neomexicanus*, M	28.1	27.6–28.6	26.8–29.6	0.76	11
	*Tibicen chiricahua*, F	29.1	28.0–30.2	28.0–30.1	0.88	5
	*Tibicen neomexicanus*, F	26.7	26.1–27.3	26.2–27.4	0.48	5
peak frequency (kHz)	*Tibicen chiricahua*	7.12	6.56–7.67	5.73–8.47	0.82	11
	*Tibicen neomexicanus*	7.27	7.02–7.52	6.07–7.81	0.45	15
amp. burst rate (bursts/s)	*Tibicen chiricahua*	54	52.4–55.6	50.5–59.3	2.32	11
	*Tibicen neomexicanus*	27.8	27.4–28.3	26.5–29.5	0.86	15
pulses per amplitude burst	*Tibicen chiricahua*	5.02	4.54–5.51	3.92–6.42	0.723	11
	*Tibicen neomexicanus*	8.34	7.70–8.98	7.25–11.33	1.066	13
phrase 1 length (s)	*Tibicen chiricahua*	1.72	1.21–2.23	0.49–2.80	0.758	11
	*Tibicen neomexicanus*	2.04	1.66–2.42	1.25–3.24	0.6	12
phrase 2 length (s)	*Tibicen chiricahua*	7.82	6.95–8.68	6.02–10.83	1.286	11
	*Tibicen neomexicanus*	6.68	5.77–7.59	4.86–10.96	1.575	14
phrase 3 length (s)	*Tibicen chiricahua*	3.75	3.10–4.39	2.37–5.82	0.963	11
	*Tibicen neomexicanus*	1.65	1.36–1.94	0.75–2.33	0.479	13

Analysis of the linear model including both species and sex as predictors of fore wing length revealed that this simple model explained much of the variation in size among the cicadas, and that the effects of both predictors were highly significant (*R^2^* = 0.805, *p* < 0.000001 for both variables). After adjusting for the size differences between males and females, the fore wings of *Tibicen neomexicensis* are, on average, about 2.9 mm shorter than those of *Tibicen chiricahua* (95% CI: 2.3–3.5). An *F*-test comparing this simple two-factor model to a model that included a (species∙sex) interaction term revealed that the two models were not significantly different (*F* = 1.202, *p* = 0.282). Therefore, the data show that for the morphometric measurements used in this study, *Tibicen neomexicensis* is significantly smaller than *Tibicen chiricahua*, and that for both species, females are significantly smaller than males. It must be noted, though, that this analysis was limited to localities for which acoustic data were available, and it is possible that these species exhibit greater variation in size across their full ranges.

##### Description and comparison of the calling songs of *Tibicen neomexicensis* and *Tibicen chiricahua*

**Figure 4.**
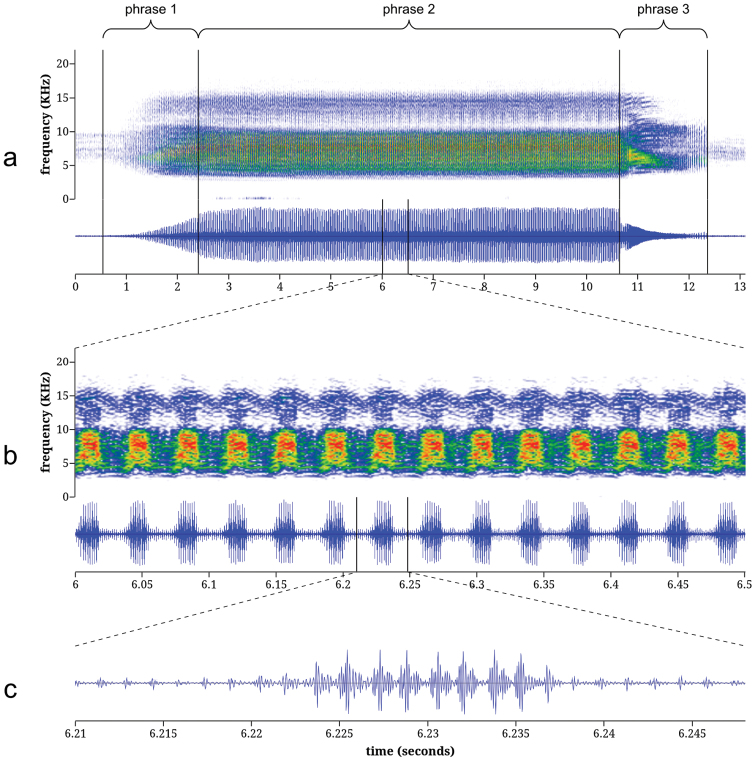
Spectrograms and oscillograms of the calling song of *Tibicen neomexicensis* sp. n.: **a** complete call **b** 0.5 seconds from the middle of phrase 2, illustrating 14 amplitude bursts **c** a single amplitude burst from **b**, illustrating the pulse structure. Spectrograms were generated using a 256-sample Fast Fourier Transform with the Hamming window function.

**Calling song of *Tibicen neomexicensis*.** The calling song of *Tibicen neomexicensis* can be divided into three phrases, each of which consists of a continuous train of pulses (Figure 4). The first phrase represents the initial increase in amplitude as the cicada begins calling and lasts an average of 2.04 seconds (95% CI: 1.66–2.42; full descriptive statistics for all acoustic parameters are given in Table 1). The second phrase is the main phrase of the call and is produced at or near maximum amplitude. This phrase lasts an average of 6.68 seconds (95% CI: 5.77–7.59) and has a mean peak frequency of 7.27 kHz (95% CI: 7.02–7.52). The first two phrases are characterized by distinctive amplitude and frequency modulations that group pulses into regular “bursts” of high amplitude. During the main phrase, these amplitude bursts are delivered at a mean rate of 27.8 bursts/s (95% CI: 27.4–28.3) and each amplitude burst consists of 8.34 pulses on average (95% CI: 7.70–8.98). The amplitude and frequency modulations are accompanied by rapid dorso-ventral movements of the cicada’s abdomen. These movements modulate frequency and amplitude by changing the acoustic properties of the sound-producing system (Pringle 1954). The third and final phrase of the call lasts an average of 1.65 seconds (95% CI: 1.36–1.94) and begins with a rapid initial drop in overall amplitude followed by a gradual decrease in amplitude until the calling song ends. During this final phrase, the amplitude and frequency modulations disappear, although the modulations sometimes briefly return as the call terminates.

**Figure 5.**
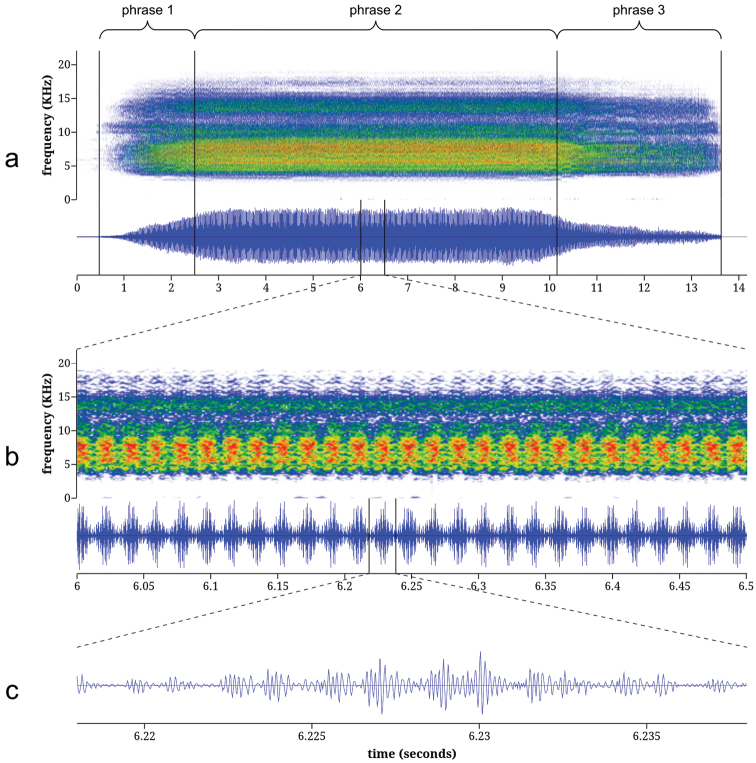
Spectrograms and oscillograms of the calling song of *Tibicen chiricahua*: **a** complete call **b** 0.5 seconds from the middle of phrase 2, illustrating 27 amplitude bursts **c** a single amplitude burst from **b**, illustrating the pulse structure. Spectrograms were generated using a 256-sample Fast Fourier Transform with the Hamming window function.

**Calling song of*Tibicen chiricahua*.** The calling song of *Tibicen chiricahua* is also naturally divided into three phrases (Figure 5). The first phrase is the initial crescendo as the call begins and lasts an average of 1.72 seconds (95% CI: 1.21–2.23). The second, main phrase of the call has a mean duration of 7.82 seconds (95% CI: 6.95–8.68) with a peak frequency of 7.12 kHz (95% CI: 6.56–7.67). The third phrase is a gradual decrescendo as the calling song terminates and lasts an average of 3.75 seconds (95% CI: 3.10–4.39). The entire call consists of an amplitude-modulated train of pulses. Pulses are grouped into high-amplitude bursts that, during the main phrase of the call, contain an average of 5.02 pulses per burst (95% CI: 4.54–5.51) and are delivered at a mean rate of 54.0 bursts/s (95% CI: 52.4–55.6).

**Comparison of calling songs.** Comparison of acoustic parameters, song structure, and physical behavior during call production verified that the calls of these two species are distinct. First, the underlying structures of the amplitude modulations of the calls differ. The mean amplitude burst rate of the call of *Tibicen chiricahua* is nearly twice that of *Tibicen neomexicensis* (54.0 and 27.8 bursts/s, respectively, *t* = 37.4, *p* < 0.000001), and the amplitude bursts of *Tibicen chiricahua* contain about 3.3 fewer pulses per burst, on average, than those of *Tibicen neomexicensis* (5.02 and 8.34 pulses/burst, respectively, *t* = 18.0, *p* < 0.000001). There was no overlap in the ranges of observed values for either of these variables. Second, the phrasal structures of the calls also differ. The phrases in the call of *Tibicen chiricahua* are defined merely by the overall pattern of amplitude changes in the call and have a relatively uniform sound quality throughout, while the third phrase of the call of *Tibicen neomexicensis* is markedly different in quality from the other two phrases, lacking the characteristic modulations of phrases one and two. Furthermore, the beginning of the third phrase in *Tibicen neomexicensis* is usually marked by an abrupt drop in amplitude, but the amplitude decreases gradually and smoothly from the second to the third phrases of *Tibicen chiricahua*. Finally, the amplitude and frequency modulations in the call of *Tibicen neomexicensis* are a result of rapid dorso-ventral movements of the abdomen during the calling song, but no such movements were apparent in the calling behavior of *Tibicen chiricahua*.

The observed mean peak frequency of the main phrase of the call of *Tibicen neomexicensis* was slightly higher than that of *Tibicen chiricahua*, although the difference was not significant (7.27 and 7.12 kHz, respectively, *t* = 0.623, *p* = 0.539). Peak calling song frequency is constrained by body size for most cicadas, with larger cicadas having lower-frequency calls (Bennet-Clark and Young 1994). Thus, given that *Tibicen neomexicensis* is smaller than *Tibicen chiricahua* but the two cicadas are not grossly dissimilar in size, it is not surprising that their peak call frequencies are similar, and even though the difference was not significant, the observed higher pitch of the call of *Tibicen neomexicensis* is consistent with the morphometric analysis.

##### Geographic distribution

**Figure 6.**
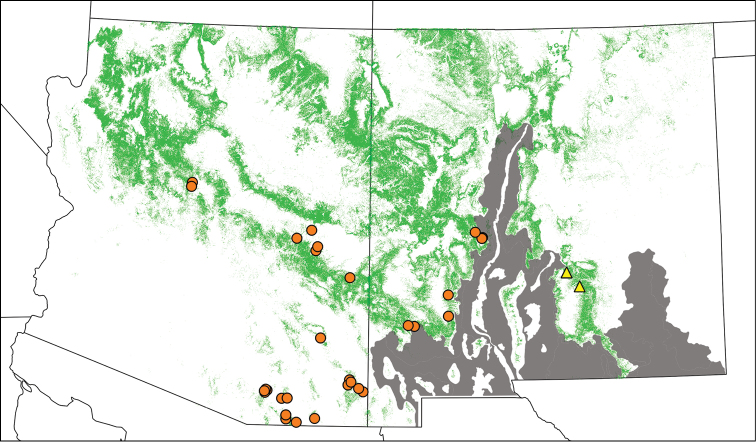
Geographic distribution of *Tibicen chiricahua* (orange circles) and *Tibicen neomexicensis* sp. n. (yellow triangles), estimated from field observations and museum specimens. Green regions indicate pinyon-juniper habitats. The gray region represents the Albuquerque Basin and Chihuahuan Desert. *Tibicen chiricahua* is also found in Mexico (Sanborn 2007).

*Tibicen chiricahua* is more widely distributed than *Tibicen neomexicensis*, ranging from central and southeastern Arizona to southwestern New Mexico (Figure 6). Although not depicted in Figure 6, *Tibicen chiricahua* is also known from Chihuahua, Mexico (Sanborn 2007). *Tibicen neomexicensis* is so far known only from the Sacramento Mountains in south-central New Mexico. All known localities for *Tibicen chiricahua* are west of the Rio Grande, while *Tibicen neomexicensis* has only been found east of the Rio Grande.

Four museum specimens, representing two unique collecting localities, could not be conclusively identified. One, a female collected June 15, 1937 in “Big Bend Park,” Brewster Co, TX (TAMU), appeared to be *Tibicen neomexicensis*. However, Phillips and Sanborn (2007) did not report any cicadas resembling *Tibicen chiricahua* in their intensive surveys of Big Bend National Park, so this record is doubtful. The other three specimens were two males and one female collected June, 1966 “near” Ciudad Cuauhtémoc, Chihuahua, Mexico (UCMC). These specimens are similar to *Tibicen neomexicensis* and *Tibicen chiricahua*, but differ in that all three have abdomens strongly marked with orange dorsolaterally. More information is needed regarding cicadas from this locality to properly determine their taxonomic status.

## Discussion

*Tibicen* is the second most diverse cicada genus in North America north of Mexico (Sanborn and Heath 2012), and recognition of *Tibicen neomexicensis* increases the number of described species in this region to 32. *Tibicen neomexicensis* belongs to the “southwestern *Tibicen* species,” an informal subgroup of *Tibicen* species that differ morphologically from the *Tibicen* cicadas common in the eastern U.S (Davis 1930). These species are only found in the western United States and Mexico.

### Diagnosis

*Tibicen neomexicensis* can be separated from all other North American species of *Tibicen* except for *Tibicen chiricahua* by the combination of its size, almost entirely black dorsal color pattern (Figure 1), and the male’s genitalia, particularly the shape of the uncus (Figure 2). Within *Tibicen*, this uncal structure is unique to *Tibicen neomexicensis* and *Tibicen chiricahua* (see Davis 1923 for a figure of the uncus of *Tibicen chiricahua*).

In the field, *Tibicen neomexicensis* and *Tibicen chiricahua* are most easily distinguished by the unique calling songs of the males. Audio recordings of both species are available as online supplementary data for this paper. To human ears, the first and second phrases of the call of *Tibicen neomexicensis* sound like a high-pitched, whiny buzz with easily discernible pulsations that correspond to the amplitude and frequency modulations. At the beginning of the third phrase, the abrupt transition to an unmodulated, uniform whine is perhaps the most aurally distinctive feature of the calling song.

In contrast, the call of *Tibicen chiricahua* sounds like a monotonous, coarse buzz that rapidly increases in amplitude during the first phrase and then slowly fades away during the final phrase. Apart from the amplitude changes in the first and third phrases, there are no obvious changes in sound quality during the course of the call.

Both male and female specimens of *Tibicen neomexicensis* and *Tibicen chiricahua* can usually also be separated by the coloration of the wing venation. In *Tibicen neomexicensis*, the anterior margin of the subcostal vein (Sc) of the fore wing is usually yellowish or at least noticeably lighter in color than the main part of the vein, which is dark black (Figure 3). In *Tibicen chiricahua*, both the vein and its anterior margin are black (Figure 3). In addition, the cubitus anterior vein (CuA) in the hind wing of *Tibicen neomexicensis* is yellow from its base to the junction with its second branch (CuA_2_), and the basal two-thirds or more of CuA_2_ is usually also yellow (Figure 3). In *Tibicen chiricahua*, these two veins tend to be mostly or entirely black (Figure 3).

Although none of these morphological characters are 100% reliable, when used in combination, they identify nearly all specimens. The color of the margin of the Sc vein is the most reliable single morphological diagnostic character. Out of nearly 200 specimens examined, only 7 might have been misidentified by the color of the Sc vein alone. The color of CuA and CuA_2_ is more variable, with some overlap between the two species, and the utility of this character seems to vary among populations of *Tibicen chiricahua*. Unfortunately, in very old specimens, the colors of the wing veins sometimes fade, making identification difficult. Fore wing length can also be used to help confirm an identification, especially when the wing vein colors are ambiguous.

### Ecology and behavior

Both *Tibicen neomexicensis* and *Tibicen chiricahua* are associated with pinyon-juniper woodlands, and neither species seems to occur in habitats where both pinyon pines (*Pinus edulis*, primarily) and junipers (*Juniperus* sp.) are absent (B. Stucky, pers. observation; Hastings and Toolson 1991). Although Davis (1925), citing a correspondence from Douglas K. Duncan, reported specimens of *Tibicen chiricahua* collected “on a high mountain plateau … devoid of any vegetation except many clumps of a large heavy grass,” he also noted that, “There is much timber around the edges of this plateau, pine, cedar, and juniper.” Overall, records of these cicadas from Arizona and New Mexico closely overlap with the distribution of pinyon-juniper forests in those states (Figure 6).

Specimen label data and field observations indicate that adults of *Tibicen neomexicensis* and *Tibicen chiricahua* emerge in early summer and are mostly gone by the end of July. The earliest record for *Tibicen chiricahua* is May 25 (in 1997) (specimen, CSUC). Although the UAIC has a specimen of *Tibicen chiricahua* from Arizona with the date recorded as “September,” the next latest collecting date is July 22 (in 1975) (UAIC), so the September date is either very unusual or in error. The majority of collecting events for *Tibicen chiricahua* were in June, and Hastings and Toolson (1991) reported that June 1–8 was approximately the middle of the adult active season of *Tibicen chiricahua* in the San Mateo Mountains of New Mexico in 1989.

Phenological data for *Tibicen neomexicensis* are much more limited, but consistent with an annual pattern similar to *Tibicen chiricahua*. The earliest record for *Tibicen neomexicensis* is for May 30 (in 2012), at the type locality (B. Stucky, pers. observation), but at this time, there were already large numbers of females ovipositing, so the cicadas must have emerged some number of days earlier. The latest record is from June 7 (in 2005) (specimen, NMSU).

The daily activity patterns of these two cicada species are also similar. Once the sun warms them sufficiently, males of both species will sing throughout much of the day, with peak calling activity occurring from about mid-day through early afternoon (B. Stucky, pers. observation; Hastings and Toolson 1991). Calling activity greatly diminishes during the late afternoon and evening.

Although the nymphal host plants of *Tibicen chiricahua* and *Tibicen neomexicensis* are not known with certainty, there is anecdotal evidence that females of these species have different oviposition preferences. Hastings and Toolson (1991) reported *Tibicen chiricahua* females ovipositing in dead pinyon pine and juniper branches. In contrast, numerous ovipositing females of *Tibicen neomexicensis* were observed at the type locality, most of which were placing their eggs in the dead, dried stems of grasses and forbs, often quite near to the ground.

Both *Tibicen chiricahua* and *Tibicen neomexicensis* are commonly found with *Tibicen duryi* Davis, another species that is specialized on pinyon-juniper habitats (Hastings et al. 1991, Kondratieff et al. 2002). At the type locality, *Tibicen neomexicensis* was also syntopic with *Okanagana bella* Davis.

### Relationship with *Tibicen chiricahua*

*Tibicen neomexicensis* and *Tibicen chiricahua* are not only extremely similar morphologically, but the shared structure of the male genitalia separates them from all other species of *Tibicen*. It therefore seems probable that *Tibicen neomexicensis* and *Tibicen chiricahua* are sister species, although a broader phylogenetic analysis of *Tibicen* is needed to confirm this.

Today, these species are apparently entirely allopatric, separated from one another by the uninhabitable Albuquerque Basin and Chihuahuan Desert. This might be a relatively recent phenomenon, though. At the time of the last glacial maximum, pinyons and junipers were widespread across much of what is today the Chihuahuan Desert (Betancourt et al. 1993, Lanner and Van Devender 1998, Thompson and Anderson 2001). As the climate changed at the end of the Pleistocene and beginning of the Holocene, pinyons and junipers retreated to higher elevations and became extirpated from modern desert regions (Betancourt et al. 1993). Moreover, climatological data and models suggest that this pattern probably repeated multiple times during the Pleistocene (Paillard 1997, Petit et al. 1999, Smith and Farrell 2005).

These habitat changes must have certainly affected the distributions of and interactions among the ancestors of modern *Tibicen neomexicensis* and *Tibicen chiricahua*. What impact, if any, this had on population divergence and speciation is unknown. However, theory predicts that secondary sexual traits can diverge rapidly in allopatry (Pomiankowski and Iwasa 1998), and if *Tibicen neomexicensis* and *Tibicen chiricahua* are sister species, it seems likely that geographic isolation caused by habitat shifts played at least some role in their evolution.

## Conclusions

Acoustic, morphometric, and behavioral data all indicate that the cicadas resembling *Tibicen chiricahua* from New Mexico’s Sacramento Mountains should be recognized as a distinct species, described here as *Tibicen neomexicensis*. In particular, analysis of audio recordings confirms that the calls of these two species have significant, consistent structural and temporal differences, which provide the simplest means for identifying these cicadas in the field.

With the discovery of *Tibicen neomexicensis*, the North American *Tibicen* are now known to encompass at least three complexes of morphologically cryptic species with distinct male calling songs: the *chiricahua* group [*Tibicen chiricahua* and *Tibicen neomexicensis*], the *dorsatus* group [*Tibicen dorsatus* (Say) and *Tibicen tremulus* Cole], and the *pruinosus* group [*Tibicen linnei* (Smith & Grossbeck), *Tibicen pruinosus* (Say), and *Tibicen robinsonianus* Davis]. A phylogeographic and divergence-time analysis of the North American *Tibicen* species based on molecular data could not only help clarify the relationship between *Tibicen neomexicensis* and *Tibicen chiricahua*, but also shed light on the broader patterns of diversification for one of the most species-rich cicada genera in North America.

The geographic ranges of these species are still rather poorly documented, especially in Mexico, where *Tibicen chiricahua* is currently known only from a single specimen (Sanborn 2007). Furthermore, compared to *Tibicen chiricahua*, few localities are known for *Tibicen neomexicensis*. Thus, additional field work is needed to clarify the distributions of these species. For *Tibicen neomexicensis*, mountain ranges near the Sacramento Mountains that also have pinyon-juniper habitats, such as the Capitan Mountains to the northeast and Guadalupe Mountains to the southeast, are obvious targets for further exploration.

## Supplementary Material

XML Treatment for Tibicen neomexicensis

## Appendix I

Calling song of *Tibicen neomexicensis* sp. n. (doi: 10.3897/zookeys.337.5950.app1) File format: Waveform Audio File Format (wav).

**Explanation note:** Recorded on May 31, 2012, at the type locality for *Tibicen neomexicensis*. Details of the recording methods are provided in the main text.

## Appendix II

Calling song of *Tibicen chiricahua* Davis. (doi: 10.3897/zookeys.337.5950.app2) File format: Waveform Audio File Format (wav).

**Explanation note:** Recorded on June 1, 2012, at the type locality for *Tibicen chiricahua*. Details of the recording methods are provided in the main text.
